# Virus-induced gene-silencing in wheat spikes and grains and its application in functional analysis of HMW-GS-encoding genes

**DOI:** 10.1186/1471-2229-12-141

**Published:** 2012-08-10

**Authors:** Meng Ma, Yan Yan, Li Huang, Mingshun Chen, Huixian Zhao

**Affiliations:** 1 State Key Laboratory of Crop Stress Biology for Arid Areas, Yangling, Shaanxi, 712100, China; 2 College of Life Sciences, Northwest A & F University, Yangling, Shaanxi, 712100, China; 3 Department of Plant Sciences & Plant Pathology, Montana State University, Bozeman, MT59717, USA; 4 USDA-ARS and Department of Entomology, Kansas State University, Manhattan, KS66506, USA

**Keywords:** Triticum aestivum, Spike, Grain, Barley stripe mosaic virus (BSMV), Virus-induced gene silencing (VIGS), Functional genomics

## Abstract

**Background:**

The *Barley stripe mosaic virus* (BSMV)-based vector has been developed and used for gene silencing in barley and wheat seedlings to assess gene functions in pathogen- or insect-resistance, but conditions for gene silencing in spikes and grains have not been evaluated. In this study, we explored the feasibility of using BSMV for gene silencing in wheat spikes or grains.

**Results:**

Apparent photobleaching on the spikes infected with BSMV:PDS at heading stage was observed after13 days post inoculation (dpi), and persisted until 30dpi, while the spikes inoculated with BSMV:00 remained green during the same period. Grains of BSMV:PDS infected spikes also exhibited photobleaching. Molecular analysis indicated that photobleached spikes or grains resulted from the reduction of endogenous *PDS* transcript abundances, suggesting that BSMV:PDS was able to induce *PDS* silencing in wheat spikes and grains. Inoculation onto wheat spikes from heading to flowering stage was optimal for efficient silencing of *PDS* in wheat spikes. Furthermore, we used the BSMV-based system to reduce the transcript level of *1Bx14*, a gene encoding for High-molecular-weight glutenin subunit 1Bx14 (HMW-GS 1Bx14), by 97 % in the grains of the BSMV:1Bx14 infected spikes at 15dpi, compared with that in BSMV:00 infected spikes, and the reduction persisted until at least 25 dpi. The amount of the HMW-GS 1Bx14 was also detectably decreased. The percentage of glutenin macropolymeric proteins in total proteins was significantly reduced in the grains of *1Bx14*-silenced plants as compared with that in the grains of BSMV:00 infected control plants, indicating that HMW-GS 1Bx14 is one of major components participating in the formation of glutenin macropolymers in wheat grains.

**Conclusion:**

This is one of the first reports of successful application of BSMV-based virus-induced-gene-silencing (VIGS) for gene knockdown in wheat spikes and grains and its application in functional analysis of the *1Bx14* gene. The established BSMV-VIGS system will be very useful in future research on functional analysis of genes contributing to grain quality and the metabolic networks in developing seeds of wheat.

## Background

Wheat (*Triticum aestivum* L.) is one of the major staple crops for the human diet. With the increase in the global population, the shortage of foods has become more and more serious. Therefore, wheat yield and quality improvement has always been the most important target in wheat breeding programs. Although conventional breeding approaches via manipulating genetic variation have been very successful in improving the agronomically important traits of cereals, the next wave of crop improvement will require much greater knowledge of gene function [1].

The rapid advance in genomics [2,3] has greatly facilitated gene isolation and manipulation, generating huge quantities of transcript and putative gene sequences (http://www.ncbi.nlm.nih.gov/guide/dna-rna/). In addition, genomes of major crops including rice, maize, and wheat have been or are being sequenced. As a result of rapid advance in structural genomics, the availability of powerful tools for gene function analysis has become a bottleneck, especially for important crops beyond the few model plant species.

In model plants such as *Arabidopsis* and rice, two methods, i.e. T-DNA knockout libraries [4] and T-DNA activation libraries [5], have significantly accelerated the speed of gene functional identification. Large collections of plants that contain T-DNA insertions have been generated. Once plants have been identified with the desired phenotypes, isolation of relevant genes is readily accomplished by finding the genomic location of the T-DNA associated with the mutant phenotype [1]. However, none of these techniques can be used in wheat due to the large genome size and very low transformation efficiency which pose a technical challenge to produce the number of transformants needed to saturate the wheat genome. An additional complication for determining gene function through the analysis of loss-of-function mutations in wheat is the fact that all cultivated varieties are polyploid; therefore in most cases, expression of homeologous genes could mask loss-of-function phenotypes resulting from the disruption of the other homeologous alleles [1].

In recent years, virus-induced gene silencing (VIGS) has been developed as an effective genetics tool for assessing gene functions in a range of dicot plant species including *Nicotiana spp.* (tobacco), *Pisum sativum* (pea), *ArabidopsisSolanum lycopersicum* (tomato) [6-9], and some monocot species *Hordeum vulgare* (barley), *Triticum aestivum* (wheat), and *Zea mays* (maize) [10-12]. VIGS was a mechanism of defense response naturally present in plants and other organisms through RNA-mediated post-transcriptional gene silencing (PTGS) to fight pathogens [13,14]. In VIGS, viruses trigger defense machinery of the hosts related to post-transcriptional gene silencing, where double stranded RNA is converted into short interfering RNAs (siRNAs). A gene of interest can be introduced into a virus vector and the recombinant virus will trigger the host defense response and both the virus genome and the endogenous mRNAs homologous to the inserted target sequence become the targets for degradation [15,16]. Silencing initiated by VIGS will spread systemically along with the siRNA [17,18], and by this method, it is possible to knockdown almost any gene of interest if a suitable vector is present for the plant species under investigation.

Many viral vectors for VIGS have been developed for various plant species [6,7,10,19-22]. However, so far, most VIGS protocols are established for gene silencing in vegetative tissues, with very limited success in reproductive and other tissues [23-29]. Gene silencing techniques for developing seeds will be very useful for studying gene functions in seed development and for seed quality improvement. For monocotyledons, two vectors, the *Barley stripe mosaic virus* (BSMV)-based vector for barley and wheat [10,11] and the *Brome mosaic virus* (BMV)-based vector for barley, rice and maize [12], have been developed and are being widely used for gene silencing in seedlings [30-32]. More recently, Pacak et al. [33] presented the first evidence of BSMV induced gene silencing (BSMV-VIGS) in the roots of a monocotyledonous plant species. To date, however, no reports have been published on BSMV-VIGS in spikes or grains of monocotyledonous species. Genes expressed in spikes or grains are likely involved in important agronomic traits such as yield, quality and disease resistance, and therefore, revealing the functions of these genes will greatly facilitate the improvement of yield, quality and pathogen resistance in cereals [34-37]. Accordingly techniques for analyzing gene functions in spikes or grains are needed for functional analysis.

High-molecular-weight glutenin subunits (HMW-GSs) are a group of endosperm storage proteins in wheat encoded by a multi-gene family. Members in this gene family possess highly homologous sequences, as found in *Glu-A1Glu-B1* and *Glu-D1* loci on the long arm of chromosome 1A, 1B and 1D, respectively [38]. The genes at the three loci share a high percentage of homologous sequences. At each locus, one x-type and one y-type subunits are encoded. The composition and amount of HMW-GSs have a profound influence on the baking quality of wheat [39,40]. HMW-GS 1Bx14 is encoded by the gene at the *Glu-B1* locus. Several studies have shown that the 1Bx14 + 1By15 subunit pair has a positive influence on the end use quality of wheat varieties [41]. More recently, the *1Bx14* gene has been isolated and sequenced (GenBank accession: AY367771.1). A *1Bx14* knockout mutant derived from an elite Chinese wheat variety Xiaoyan 54 through chemical mutagenesis was generated and characterized, providing an opportunity for identifying the biological function of *1Bx14*[42]. We tried to generate transgenic lines with a *1Bx14* over-expressed construct by microprojectile bombardment to assess the gene function, but introduction of this gene led to silence of several other homologous endogenous genes in the common wheat cv. Mianyang 19 [43].

**Figure 1:**
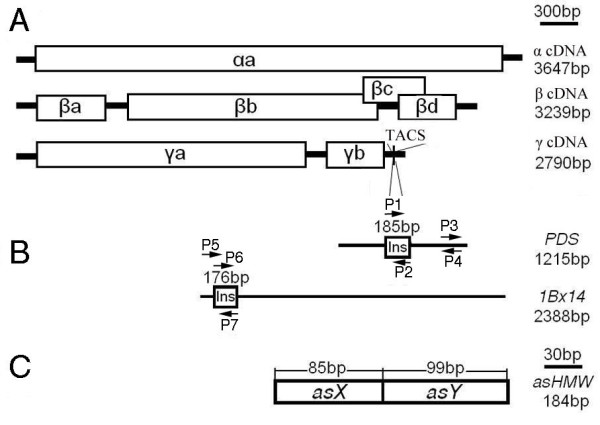
** Schematic organization of the*Barley stripe mosaic virus*(BSMV) genomes and the inserts used for BSMV virus-induced-gene-silencing (VIGS).** (**A**) Genomic organization of the three BSMV components *α*, *β* and *γ* drawn to scale of 300 bp. Open reading frames are indicated by boxes. The TA cloning site (TACS) designed for direct cloning of PCR products is positioned after the stop codon of the *γb* gene. (**B**) Schematic representation of full-length phytoene desaturase gene (*PDS*) and *1Bx14* cDNAs (black line) drawn to the same scale as above. The 185-bp fragment of *PDS* and the 176-bp fragment of *1Bx14* cDNAs indicated by boxes are inserts for BSMV-VIGS. P1 and P2, P6 and P7 are primer sets for generating the VIGS construct BSMV:PDS and BSMV:1Bx14, respectively. P3 and P4, P5 and P7 are primer sets for quantitative real-time RT-PCR (qRT-PCR) analysis of *PDS* and *1Bx14* transcripts, respectively. (**C**) Schematic representation of a 184-bp insert fragment of artificially synthesized sequence (*asHMW*) with one 85-bp sequence that is identical to most x-type glutenin subunit genes (*asX*) and one 99-bp sequence that is identical to most y-type genes (*asY*), drawn to the scale of 30 bp.

Our long term goal is to elucidate the effect of specific proteins on wheat grain quality and end use. In this study, we first tested the possibility of employing BSMV vector to silence genes in wheat spikes or grains with the marker gene *PDS* encoding phytoene desaturase*,* an enzyme required for the biosynthesis of carotenoid pigments that protect chlorophyll from photobleaching. Silencing of *PDS* can be visualized as white streaks resulting from photobleached chlorophyll [10]. After optimizing the locations and stages for VIGS in wheat spikes and grains, we chose to silence the *HMW-GS 1Bx14* gene because the protein products have been shown by our group to participate in forming larger glutenin polymers and greatly contribute to dough strength [44]. We also chose to silence the entire family of *HMW-GS* genes related to wheat gluten quality for testing the feasibility of BSMV-VIGS for functional analysis in wheat grains (Figure 1). The results in this study indicate that BSMV-VIGS system is a useful tool for functional analysis of genes expressed in wheat spikes and grains.

## Results

### BSMV is able to induce gene silence in wheat spikes/grains

To examine whether BSMV vector can induce gene silencing in wheat spikes and grains, we used the BSMV vectors carrying a 185-bp fragment of barley *PDS* gene (designated as BSMV:PDS) [11,45] for the initial silencing experiment. Two winter wheat, cvs Xiaoyan 6 and Shaanyou 225, and a spring wheat, cv. Ningchun 16, were used in this experiment. To test the infectivity and the efficacy of BSMV-VIGS in all three wheat cultivars, we conducted a preliminary experiment at the seedling stage to silence the *PDS* gene. We inoculated ten seedlings of each wheat cultivar with BSMV:PDS and BSMV:00 (for only the BSMV genome without the target gene), respectively, onto the 2^nd^ fully expanded leaves of 10-day-old seedlings. Mosaic and chlorotic stripes were observed on the tip portions of the third leaves of all inoculated plants about 7 days post inoculation (dpi). Only the BSMV:PDS inoculated plants (10 out of 10) showed photobleaching starting from the base areas of the third leaves and extending throughout the length of the fourth leaves during 10 ~ 30 dpi. Photobleaching was very rarely seen in the fifth leaves of all three cultivars (data not shown). No photobleaching was observed in the plants infected with BSMV:00. These observations indicated that the BSMV:PDS construct could systemically induce gene silencing in all three cultivars and efficiently induce *PDS* gene silencing in these cultivars.

**Figure 2:**
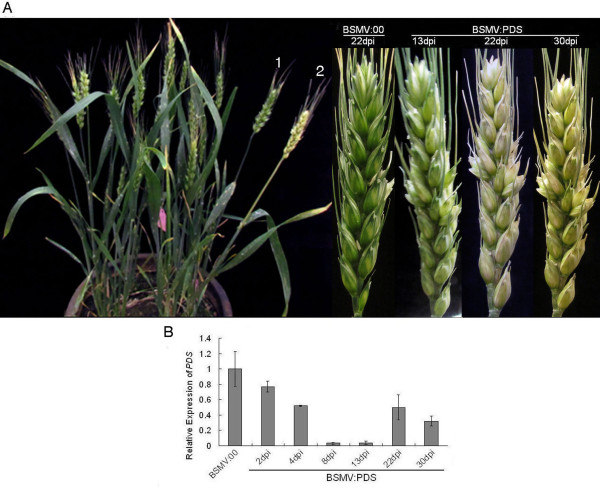
** Silencing endogenous phytoene desaturase gene (*PDS*) in wheat spikes.** Wheat cv. Xiaoyan 6 was infected with in vitro transcribed RNAs representing the *α*, *β* and *γ* RNAs of BSMV:00 or *α*, *β* and *γPDS* RNAs of BSMV:PDS onto the spikes at the heading stage. (**A**) Left photograph indicates the phenotypes of wheat plants respectively infected with BSMV:00 (1) and BSMV:PDS (2) at 22 days post-inoculation (dpi); the right one is the spikes photographed at 13dpi, 22dpi and 30dpi, respectively. The spikes shown are representative of different treated plants. (**B**) The relative expression levels of *PDS* in the wheat spikes inoculated with BSMV:PDS at 2, 4, 8, 13, 22 and 30 dpi are determined by quantitative real-time PCR (qRT-PCR) (normalization to *actin*) compared the BSMV:00 control inoculated with BSMV:PDS. Each column represents the mean of three samples, and error bars indicate the standard deviation.

In order to investigate the effectiveness of BSMV-VIGS in wheat spikes, endogenous *PDS* was again selected as a target to silence. We inoculated ten plants of each wheat cultivar on their spikes at heading stage by rubbing each spike five times with BSMV:PDS and BSMV:00, respectively. Unlike the progress of BSMV:PDS infection observed on leaves of wheat seedling, no mosaic symptoms and chlorosis were observed on wheat spikes by 13 dpi; only some ring-like or stripe symptoms were visible on the awns of some infected spikes. The symptoms of wheat spikes infected with BSMV:PDS were indistinguishable from those infected with BSMV:00 at 13 dpi or earlier. However, photobleaching on the spikes infected with BSMV:PDS appeared after 13dpi, and persisted until 30dpi, while the spikes inoculated with BSMV:00 remained green during the same period (Figure 2A). Nine of the ten plants (90%) inoculated with BSMV:PDS showed photobleaching throughout the entire wheat spike (Table 1), and the photobleaching persisted until 30 dpi (Figure 2A).

**Table 1:** **Effectiveness of*PDS*silencing in the spikes of Xiaoyan 6 plants inoculated with BSMV:PDS at three different development stages**

**Development stage**	**Inoculation location**	**No. of plants inoculated**^**a**^	**No. of plants with photobleaching phenotypes**^**b**^	
			**Partial photobleaching**	**Complete photobleaching**
Booting	Flag leaf	10	8	1
	Spike	10	2	0
Heading	Flag leaf	10	4	4
	Spike	10	0	9
Flowering	Flag leaf	10	0	0
	Spike	10	0	10

^a^ This experiment was conducted with three independent biological replicates, ten plants were inoculated for each condition.

^b^ Numbers of plants with partial or complete photobleaching were reproducible in three biological replicates.

To confirm that the observed photobleaching was the result of *PDS* silencing, we conducted quantitative real-time PCR (qRT-PCR) to measure the *PDS* transcript abundances in the spikes inoculated with BSMV:PDS at different time points. Surprisingly, a 25% reduction in *PDS* transcript abundance was detected as early as 2 dpi, but a remarkable reduction of *PDS* was detected between 8–13 dpi. From 4–30 dpi, the *PDS* mRNA level in spikes treated with BSMV:PDS was only about 60%, or lower than the level in spikes with BSMV:00 treatment (Figure 2B). The most significantly bleached spikes were seen at 22 dpi, much later than the reduction of the *PDS* transcript level.

**Figure 3:**
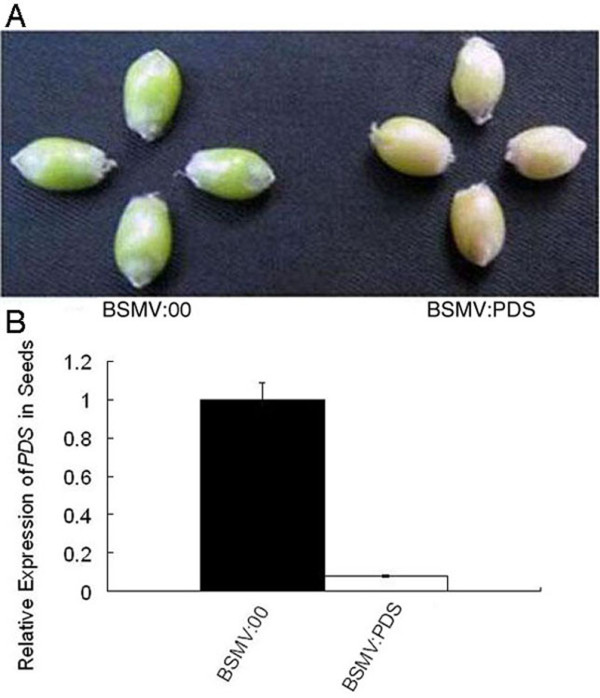
** Silencing phytoene desaturase gene (*PDS*) in the grains of wheat cv. Xiaoyan 6 by BSMV-VIGS.** (**A**) Left is four grains from a BSMV:00 inoculated spike and right is grains from a BSMV:PDS inoculated spike at 22 days post-inoculation (22dpi). The grains shown are representatives of ten different treated plants. (**B**) Transcript levels of *PDS* in these grains (Figure 3A) determined by quantitative real-time PCR (qRT-PCR).

The effectiveness of VIGS in wheat grains was then investigated. Grains from BSMV:PDS infected spikes appeared photobleached only when their glumes were taken off and the grains were exposed directly under bright light. The phenotypes of the grains from the heads infected with BSMV:PDS or BSMV:00 at 22 dpi are shown in Figure 3A with cultivar Xiaoyan 6 as a representative. Chlorosis occurred in the grains of BSMV:PDS infected heads at 22 dpi, while the grains of BSMV:00 inoculated control plants remained green, suggesting photobleaching of these seed coats was due to the silencing of the *PDS* gene. To confirm this, transcript abundances of the *PDS* gene in the photobleached and non-photobleached grains were measured by qRT-PCR. The level of *PDS* transcript abundance in the chlorotic grains was reduced by 92% (P<0.01), compared with the green ones (Figure 3B).

### Optimal conditions for efficient gene silencing in wheat spikes and grains

Similar to leaf-rub inoculation applied to wheat seedlings [1,11], spike-rub inoculation on the spikes at heading stage was also effective for gene silencing in spikes and grains. Syringe-injection inoculation onto the spikes at heading stage was also tested, but this method failed to get results as consistent as spike-rub inoculation. Therefore, spike-rub inoculation was used in all silencing assays later. Because photobleached phenotypes of the spikes or grains in BSMV:PDS inoculated plants exhibited a consistent correlation with the reduction of *PDS* transcript abundances in our experiments, photobleaching was used as an indicator of *PDS* gene silencing in wheat spikes and grains.

**Figure 4:**
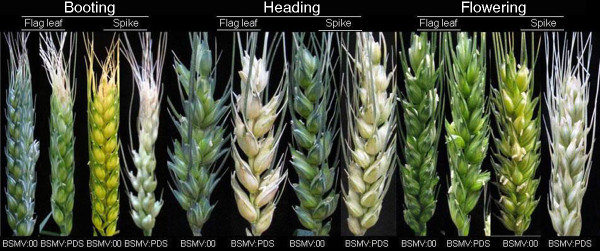
** Phenotypes of the spikes from wheat cv. Xiaoyan 6 inoculated with BSMV:PDS or BSMV:00 constructs.** The spikes shown are representatives of different treated plants and all spikes were collected at 22dpi. Booting, Heading and Flowering above indicate the inoculation stage. Flag leaf or Spike on the top of each spike indicates the inoculation location. BSMV:PDS or BSMV:00 at the bottom indicates inoculation with BSMV:PDS or BSMV:00 constructs.

To optimize conditions for efficient and stable silencing of genes in wheat spikes and grains, inoculation location and stage were determined by inoculating BSMV on flag leaves or spikes of ten plants for each wheat cultivar at three different development stages, i.e. booting, heading and flowering, respectively, by rubbing the in vitro synthesized BSMV RNAs three times on a flag leaf or five times on a spike. For simplicity, only the winter wheat cv. Xiaoyan 6 and the spring wheat cv. Ningchun 16 were tested at three different stages, and at each stage, the experiment included three independent biological replicates. As shown in Figure 4, at 22 dpi, photobleached spikes were observed in BSMV:PDS infected Xiaoyan 6 plants if BSMV:PDS was inoculated onto spikes at booting, heading or flowering stages. But if BSMV:PDS was inoculated onto flag leaves, photobleaching spikes appeared only in wheat plants inoculated at heading stage. These results were reproducible in three biological replicates. Figure 4 shows three reproducible phenotypes with different degrees of photobleaching, i.e. green spikes (no photobleaching), partially photobleached spikes (partial photobleaching) and uniform highly photobleached spikes (complete photobleaching). Effectiveness of BSMV-induced *PDS* silencing in wheat spikes was strongly influenced by the location of the inoculation and the stage of the plant when the inoculation was done. From heading to flowering stage, 90% (9/10)~100% (10/10) of plants inoculated with BSMV:PDS onto spikes exhibited highly uniform photobleaching (Table 1). Similar results were also observed in wheat cv. Ningchun 16 (data not shown). We also attempted inoculation onto wheat spikes 10 days after flowering, but failed to achieve silenced phenotypes.

Taken together, inoculation onto wheat spikes from heading to flowering stage was optimal for efficient silencing of *PDS* in wheat spikes.

### Evaluation of biological function of the *1Bx14* gene in gluten network formation in wheat grains using BSMV-VIGS

After optimizing the BSMV-based VIGS system in wheat spikes/grains, we tried to explore the usefulness of the system for functional analysis of genes expressed in wheat grains. The *1Bx14* gene (GenBank accession number AY367771.1) was chosen as a gene of interest tested in two wheat cultivars carrying *1Bx14*, Xiaoyan 6 and Shaanyou 225. To make gene silencing as specific as possible, a 176-bp fragment of *1Bx14* from the coding region (corresponding to the region of 98 to 273-bp downstream of the start codon) was inserted into the TA cloning site of the BSMV *γ* vector to generate the construct BSMV:1Bx14 (Figure 1A and 1B). The 176-bp insert fragment possesses approximately 60–82% identity to the DNAs of the *HMW-GS* gene *1Ax1, 1By15, 1Dx2* and *1Dy12* in wheat cvs Xiaoyan 6 and Shaanyou 225 (Additional file 1).

**Figure 5:**
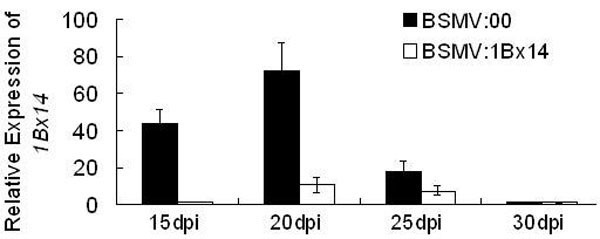
** The relative expression of*1Bx14*in the grains of Xiaoyan 6 inoculated with BSMV:1Bx14 or BSMV:00.** The grain samples were collected at 15, 20, 25 and 30 dpi for RNA isolation. The relative expression of *1Bx14* in all the grain samples was determined by quantitative real-time PCR (qRT-PCR), and normalized with *actin*. Each column represents the mean of three samples, and error bars represent the standard deviation.

**Figure 6:**
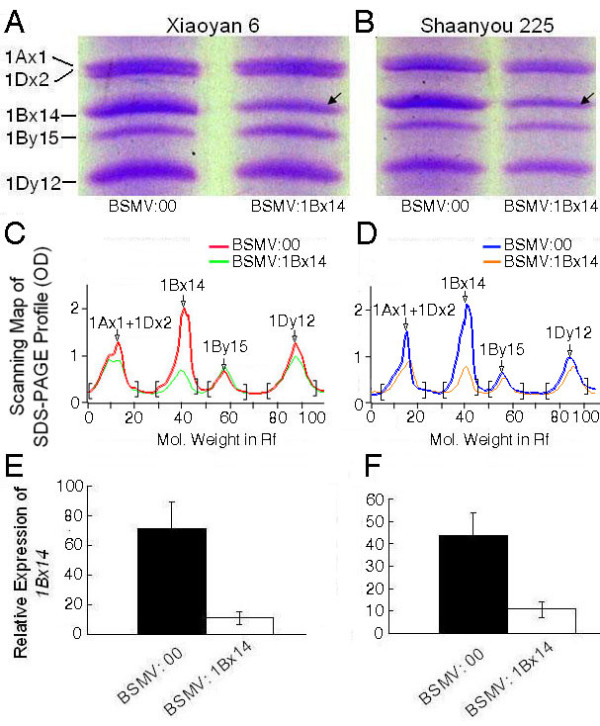
** The protein and mRNA levels of*1Bx14*in the grains of BSMV:1Bx14 infected spikes.** Grain samples were collected at 20dpi from each wheat cv. infected with BSMV:1Bx14 or BSMV:00, and each of the grains was divided into two parts, one used for Sodium dodecyl sulphate-polyacrylamide gel electrophoresis (SDS-PAGE) and the other for quantitative real-time PCR (qRT-PCR) analysis. (**A**) and (**B**) SDS-PAGE of HMW-GSs in the grain from cultivars Xiaoyan 6 or Shaanyou 225 inoculated with BSMV:00 or BSMV:1Bx14, respectively. Arrows indicate the reduced HMW-GS 1Bx14. (**C**) and (**D**) The scanning profiles of SDS-PAGE maps shown in Figure 6 and 6B. (**E**) and (**F**) QRT-PCR analysis of the relative expression of *1Bx14* in the same grain as in Figure 6A or 6B.

Ten plants of each wheat cultivar were inoculated with BSMV:00 and BSMV:1Bx14 respectively on the spikes at flowering stage. The grains in the middle of the infected spikes were collected at 15, 20, 25 and 30 dpi for RNA isolation. Transcript abundances of *1Bx14* were measured in all the grain RNA samples by qRT-PCR. The data showed that the transcript levels of *1Bx14* decreased by about 97% at 15 dpi and 85% at 20 dpi in the grains of BSMV:1Bx14 infected spikes compared with that of the BSMV:00 infected control. *Actin* was used as an internal control. The reduction persisted until at least 25 dpi (Figure 5). These data were similar to the results of *PDS* silencing described above. The amount of HMW-GS 1Bx14 of the *1Bx14*-silenced grains of Xiaoyan 6 and Shaanyou 225 was detectably decreased on Sodium dodecyl sulphate-polyacrylamide gel electrophoresis (SDS-PAGE) (Figure 6A and 6B). The scanning profiles of the SDS-PAGE gels shown in Figure 6 A and 6B shows the quantitative reduction of protein level; i.e. the peak of HMW-GS 1Bx14 in the *1Bx14*-silenced grains was about 64% reduced in cultivar Xiaoyan 6 (Figure 6C) and 67% in Shaanyou 225 (Figure 6D) compared with that in the controls, respectively. The protein levels of *1Bx14* were not reduced as much as the mRNA levels (Figure 6 E and 6 F).

To evaluate the impact of *1Bx14* knockdown in the formation of wheat gluten network, total proteins (TP), total glutenin polymer(TGP) and glutenin macropolymeric protein (GMP) of the grains from BSMV:Bx14 and BSMV:00 infected spikes were extracted and quantified by micro-Kjeldahl method, respectively. The quantity of GMP, TGP or TP in the grains of *1Bx14*-silenced plants were significantly decreased, compared with that in BSMV:00 infected controls in both Xiaoyan 6 and Shaanyou 225. The ratio of GMP to TP or the percentage of GMP in TP was significantly reduced (Table 2), indicating that HMW-GS 1Bx14 is one of the major components participating in the formation of glutenin macropolymers in wheat grains. These results are consistent with our previous conclusion, suggesting the usefulness of BSMV-VIGS system for the functional analysis of genes expressed in wheat grains.

**Table 2:** **The mean values of TP, TGP and GMP in the grains from*1Bx14*-silenced or control plants (infected with BSMV:00)**

**Cultivar**	**BSMV Construct**	**TP (%)**	**TGP (%)**	**GMP (%)**	**GMP/TP (%)**
Xiaoyan 6	BSMV:00	16.50 ± 0.06A	9.23 ± 0.07A	8.34 ± 0.06A	50.55 ± 0.51A
	BSMV:1Bx14	15.25 ± 0.04B	8.41 ± 0.08B	6.82 ± 0.12B	44.73 ± 2.93B
Shaanyou 225	BSMV:00	17.74 ± 0.07A	9.58 ± 0.10A	7.95 ± 0.04A	44.81 ± 1.89a
	BSMV:1Bx14	15.18 ± 0.13B	8.50 ± 0.11B	6.49 ± 0.09B	42.73 ± 0.75b

The mean values resulted from three independent biological replicates.

TP, TGP and GMP indicate total proteins, total glutenin polymers, and glutenin macropolymeric proteins, respectively.

Means with different capital letters indicate significantly different at P < 0.01 level.

Means with different small letters are significantly different at P < 0.05 level.

### Silencing of all *HMW-GS* gene family members in wheat grains via BSMV-VIGS system

The successful silencing of the *1Bx14* gene by the BSMV-VIGS system encouraged us to silence all the *HMW-GS* gene family members in wheat grains. A BSMV-derived construct carrying a 184-bp fragment of artificially assembled sequence (*asHMW*) was developed and named as BSMV:HMW. The 184-bp fragment contained one 85-bp sequence highly homologous to most of the x-type glutenin subunit genes (*asX*) and one 99-bp sequence identical to most of the y-type glutenin subunit genes (*asY*) (Figure 1C). Nucleotide sequence alignment of the *asX* or the *asY* with the corresponding elements in other x-typeor y-type *HMW-GS* genes in wheat was shown in Additional file 2.

**Figure 7:**
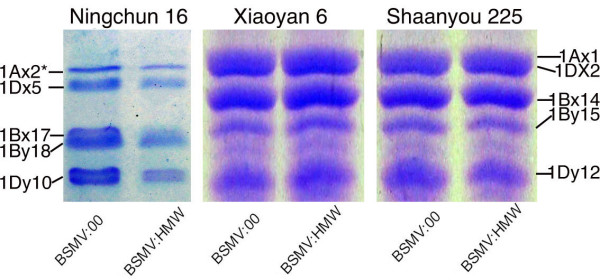
** The HMW-GS in the grains of BSMV:HMW or BSMV:00 infected wheat spikes analyzed by SDS-PAGE.** All three wheat cultivars Ningchun 16, Xiaoyan 6 and Shaanyou 225 were used in this experiment. Grain samples were collected at 30dpi from wheat plants of each cultivar infected with BSMV:1Bx14 or BSMV:00.

All three wheat cultivars mentioned above were tested in this experiment. Ten plants of each wheat cultivar were inoculated with BSMV:HMW or BSMV:00 on the spikes at flowering stage. Silencing of *HMW-GS* genes was monitored by SDS-PAGE on protein levels due to the lack of primers specific to individual target genes for real-time PCR. As shown in Figure 7, the amount of each x-type or y-type HMW-GS extracted from the seeds of the BSMV:HMW infected spikes of Ningchun 16 showed detectable decrease compared with the BSMV:00 treated spikes, indicating that all the *HMW-GS* genes 1Ax2*, *1Bx17*, *1By18*, *1Dx5* and *1Dy10*, were down-regulated in the seeds of BSMV:HMW infected spikes of Ningchun 16. However, in the seeds of BSMV:HMW infected spikes of Xiaoyan 6 and Shaanyou 225, no apparent reduction in amount of HMW-GS was observed, suggesting that all the *HMW-GS* gene family members were not efficiently silenced.

## Discussion

Despite the widespread use of VIGS in seedling plants, its application in reproductive tissues of plants is rather limited. In the review by Scofield & Nelson [46], the authors mentioned that VIGS could be achieved in the flag leaves and floral organs by inoculating upper leaves of older wheat plants with the BSMV RNAs, citing unpublished data without further details or documentation. In wheat, to date, there is no report of the application of VIGS in developing seeds. In this study, we report, for the first time, the successful application of BSMV-based VIGS for gene knockdown in wheat spikes and grains and its application in functional analysis of HMW-GS-encoding gene *1Bx14*. Unlike *Cucumber mosaic virus* (CMV)- or *Apple latent spherical virus* (ALSV)-vector for VIGS in soybean (*Glycine max*), with which inoculation onto the first leaf at the seedling stage or two cotyledons at emergence stage of soybean plants could effectively lead to gene silence in seeds [28,29], inoculation with BSMV:PDS recombinant virus onto the first leaf of wheat or barley plants at the seedling stage led to the most efficient *PDS* silencing in the third leaf, but the silence effect largely disappeared before the onset of flowering in nearly all plants. Occasionally, some degree of photobleaching was observed in the flag leaf and some of the heads [47]. When barley or wheat were infected with the BSMV:PDS construct, the duration of silencing lasted only about 4 weeks post-inoculation [11,47].

Considering both the duration of effective BSMV-based VIGS and the temporal and spatial expression of some genes related to wheat quality or yield, the optimal inoculation conditions for VIGS in wheat spikes and grains are needed to be determined for efficient gene silencing in wheat productive organs. In this study, we established a protocol for effective knockdown of genes expressed in wheat spikes and developing seeds using BSMV-based VIGS. We found that efficiency of BSMV-induced gene silencing varied greatly with the locations and the stages of inoculation, and inoculation with BSMV on wheat spikes from heading to flowering stage by spike-rubbing five times showed the most effective silencing of *PDS* in wheat spikes (Table 1). Since gene silencing with BSMV-based VIGS vector is generally transient, inoculation on spikes at the appropriate time is crucial for knockdown of genes expressed only in developing seeds.

Compared with BSMV-VIGS experiments with barley or wheat seedlings, the main visual differences distinguishing BSMV-VIGS in wheat spikes are viral symptoms, the phenotypes of plants with gene silencing, and the time period of the effect. Wheat plants inoculated with BSMV:PDS at the seedling stage exhibited patches of lesion in the leaf above the inoculated one[11]. However, for wheat plants infected with BSMV:PDS onto spikes, only ring-like or stripe symptoms appeared on the awns of infected spikes. As for the phenotype of *PDS* silencing, both leaves and spikes showed apparent photobleaching. However, photobleaching in the leaf often did not cover the entire width of the leaf and was often confined to narrow stripes parallel to the leaf veins [10], while photobleaching in the spike extended to the entire spike (Figure 2A and Figure 4). With regard to the effective time period of BSMV-VIGS, evident photobleaching initially appeared in the third and fourth leaves of the barley seedlings by 7 dpi, and leaves that emerged after 21 dpi were unlikely to have photobleaching when BSMV:PDS inoculation was done on the first leaves [10]. However, our results showed that, when wheat spikes were infected with BSMV:PDS, visual photobleaching appeared on the spikes after 13 dpi, and persisted until 30 dpi. The qRT-PCR data revealed that *PDS* transcript abundances in BSMV:PDS infected spikes reduced by about 25% at 2 dpi, 95% between 8 ~ 13 dpi, and 45% at 22 dpi, compared with those in spikes inoculated with BSMV:00 (Figure 2B). But the most remarkable photobleaching was observed at 22 dpi, much later than the reduction of *PDS* transcript. The reduction in *PDS* mRNA levels began earlier and lasted longer than the period of photobleaching (Figure 2)**.** Notably, *PDS* transcript abundances in BSMV:PDS infected spikes were prone to increase at 22 dpi and 30 dpi. This is due to the instability of recombinant BSMV:PDS leading to deletion of *PDS* sequences from the virus vector (data not shown). These conditions were also reported previously by Bruun-Rasmussen et al. [47].

After optimization of conditions, we also successfully silenced the gene *1Bx14* that encodes storage protein in wheat endosperm. The correlated reduction in the levels of the mRNA and the protein of *HMW-GS 1Bx14* suggested VIGS-treatment can effectively knockdown genes expressed in developing seeds (Figure 6). Notably, the reduction in mRNA-level and in protein level of *1Bx14* in Figure 6 was not comparable. We speculate that the reason is that the protein abundances shown in Figure 6A and 6B were the consequence of about a 10-day-accumulation because *HMW-GS* genes initially expressed 9~12 days after flowering, while the transcript abundances shown in Figure 6E and 6 F were the mRNA level of *1Bx14* in the *1Bx14*-silenced grains at 20dpi due to the short lifetime of mRNA. The peak of gene silencing induced by BSMV was at 20dpi according to qRT-PCR results in the BSMV:1Bx14 time course study (Figure 5).

However, when silencing the entire *HMW-GS* gene family in wheat grains with the BSMV:HMW construct, the results were not the same among the three wheat cultivars. All the *HMW-GS* genes 1Ax2*1Bx171By181Dx5 and *1Dy10* in spring wheat cv. Ningchun 16 were detectably down-regulated in the seeds of BSMV:HMW infected spikes, but no apparent reduction was detected in the seeds from BSMV:HMW infected spikes of cultivars Xiaoyan 6 and Shaanyou 225. Scofield et al. [11] also reported that only 21% of the wheat plants and 16% of the barley plants displayed any detectable photobleaching when infected with the BSMV:PDSas construct carrying an 80-bp PDS4as derivative fragment, and no photobleaching was observed in any of the plants infected with the 40-bp PDS4as derivative. The sizes of the inserts greatly affect the efficiency of BSMV induced gene silence. Therefore, we think that the inconsistency among the three wheat cultivars for silencing *HMW-GS* gene combinations may be due to the sizes of inserts--*asX* (85-bp) plus *asY* (99-bp) were not long enough for induction of efficient silencing.

It has been postulated based on early genetic studies [34,40] that the composition and the amount of HMW-GS in the grains can greatly affect gluten strength and thereby the end use qualities of both tetraploid and hexaploid wheat. The observation of significant alteration in the structure of gluten complex and the processing properties of wheat grains in a transgenic line that over-expresses a single HMW glutenin subunit reinforces this postulation [48]. However, more systematic analyses of the direct effects of *HMW-GS* genes on grain properties are difficult due to technical difficulties in development of an ideal transgenic line that has the target *HMW-GS* gene over-expressed without affecting other endogenous genes’ expression in wheat grains. There have been many efforts in developing transgenic wheat lines to confirm the function of *HMW-GS* genes, but most of them could not reach the final stage due to very low transformation efficiency and un-intended side effects in the transgenic wheat lines generated. For example, introduction of a construct overexpressing the gene *1Ax1* into a commercial spring bread wheat resulted in the inactivation of the 1Ax2* allele. Transformation of constructs overexpressing both *1Ax1* and *1Dx5* resulted in the silencing of all other HMW glutenin subunits [49]. The technical difficulties and phenotypic complication associated with transgenic approaches make the development of VIGS-based techniques to silence genes in grains more useful and urgent. In this study, we successfully silenced the *1Bx14* gene, which is highly expressed in seeds of wheat cvs Xiaoyan 6 and Shaanyou 225 (Figure 6E and 6B). Silencing of *1Bx14* led to a significant decrease in the quantity of GMP and the percentage of glutenin macropolymeric protein in total proteins (GMP/TP) of the grains (Table 2), indicating that HMW-GS 1Bx14 plays very important roles in the formation of glutenin macropolymers (or wheat gluten network). By using purified HMW-GS 1Bx14 as a supplement to base flour to make dough and test its functional properties, our group found that incorporation of the HMW-GS increased the quantity of glutenin macropolymeric protein (GMP) in the dough and enhanced the dough strength [44]. The present results further support this conclusion. Unexpected was that a reduction of 7%~9% in the total protein content was detected in grains of *1Bx14*-silenced plants, although an individual glutenin subunit accounts for only about 2% of total grain proteins. The effect of *1Bx14* silencing on total proteins needs to be further investigated. To our knowledge, this is the first report of knockdown genes expressed in wheat developing seeds with recombinant BSMV. The establishment of this effective approach for gene knockdown in developing seeds will allow us to dissect the genetic pathways that control seed development, grain quality and pathogen defense in grain tissue of hexaploid wheat; it will also provide an important reference for silencing genes in spikes or grains of other monocotyledonous species by VIGS.

## Conclusions

We first demonstrated the feasibility of using BSMV for gene silencing in wheat spikes or grains with *PDS* as a marker gene. We determined that inoculation with BSMV onto wheat spikes from heading to flowering stage by spike-rub five times was the optimal condition for silencing *PDS* in wheat spikes. In addition, we successfully silenced *HMW-GS* gene *1Bx14* expressing in the developing seeds through the established BSMV-VIGS approach. The BSMV-VIGS system in spikes and grains will be very useful in future research on functional identification of genes contributing to grain quality and controlling the metabolic networks in seed development of wheat.

## Methods

### Plant material

Three hexaploid wheat (*T. aestivum* L.) cultivars, two winter wheat cvs Xiaoyan 6 and Shaanyou 225, and one spring wheat cv. Ningchun 16, were used in the experiments. The two winter wheats are elite cultivars with good quality, and both possess identical HMW glutenin subunit genes*,* i.e. *1Ax1, 1Bx14, 1By15, 1Dx2* and *1Dy12*, while Ningchun 16 has HMW glutenin subunit genes 1Ax2*, 1Bx17, 1By18, 1Dx5 and *1Dy10*. For VIGS experiments, all three cultivars were grown in a greenhouse with a day/night temperature regime of 20–25°C/15–18°C, a light period of 16 h/8 h day/night, regulated with supplementary light, and watered as needed. The two winter wheat cultivars were exposed to a temperature of 4°C for 40 days to achieve complete vernalization, while the spring wheat cultivar was exposed to 4°C for 14 days at the two leaves stage. The temperature of the greenhouse was kept at a constant 22°C after the booting stage of the three cultivars.

### Construction of BSMV-derived vectors

The BSMV vectors utilized in these experiments were obtained from Dr. Andrew O. Jackson at UC Berkeley [50]. The γ vector was reconstructed to include PCR-ready cloning sites following a protocol modified from Holzberg et al. [10]. The plasmids utilized in the experiments of silencing *PDS* in wheat spikes/grains were described in Campbell [45]. In details, the BSMV γ vector was digested with *Not*I/*Pac*I and inserted the sequence of GGCCCCACTCATGACATGGCGTTAGCCATGGGAAGCTTGGAT, including two *Xcm*I restriction sites. The modified γ vector (named the γ PCR vector) was linearized with restriction enzyme *Xcm*I to produce a TA cloning site for direct cloning of PCR products. Since a BSMV *γ* RNA construct carrying a 185-bp fragment of the barley (*Hordeum vulgare* L.), the *PDS* gene in antisense orientation (BSMV:PDS4as) induced optimal silencing of *PDS* in wheat leaves as described previously [11], we used the BSMV:PDS4as for initial testing under our experimental conditions. For simplicity, the BSMV:PDS4as construct was named as BSMV:PDS and the BSMV-derived construct with no insert as BSMV:00 in our experiments.

The BSMV construct utilized to silence the *1Bx14* gene, which carrying a 176-bp fragment of *1Bx14* cDNA, was designed as BSMV:1Bx14 and constructed as follows: The 176-bp fragment of *1Bx14* from the coding region (corresponding to the region of 98 to 273-bp downstream of the start point of translation) was generated by PCR amplification from the plasmids containing the cloned *1Bx14* gene with the forward primer P6:5'- GCGAGCTCCGGAAGCGCG-3' and reverse primer P7: 5'- CGAAGGCGTAGTCTCGCTGGGG-3', and inserted into the *γ* vector (Figure 1A and 1B). The analysis of the identity between the 176-bp fragment and the full-length of *HMW-GS* gene *1Ax1, 1Dx2, 1Bx14, 1By15* and *1Dy12* in wheat cultivars Xiaoyan 6 and Shaanyou 225 was conducted with the DNAMAN software (http://www.lynnon.com).

The BSMV construct carrying a 184-bp fragment of an artificially assembled sequence (*asHMW*) with one 85-bp sequence identical to most x-type glutenin subunit genes (*asX*) and one 99-bp sequence identical to y-type glutenin subunit genes (*asY*), named as BSMV:HMW, was generated (Figure 1C) and used to silence the entire *HMW-GS* gene family in wheat grains. The BSMV:HMW was constructed as follows: The fragment containing an 85-bp fragment of *1Bx14* from the encoding region (corresponding to the region of 296 to 380-bp downstream of the start codon) and a 99-bp fragment of *1By16* (GenBank accession number EF540765.1) from the coding region (corresponding to the region of 798 to 896-bp downstream of the start codon) were synthesized (by Sangon, Shanghai, China), and inserted into the *γ* vector. The 85-bp sequence shared a 90.59–100% similarity to the corresponding elements in all x-type *HMW-GS* genes and the 99-bp sequence shared a 94.95–100% similarity to those in all y-type *HMW-GS* genes (Additional file 2).

### In vitro transcription of viral RNAs and plant inoculation

The procedures for in vitro transcription of viral RNAs were the same as described by Scofield et al. [11]. In vitro synthesized BSMV RNAs were rub-inoculated onto flag leaves or spikes at three development stages, i.e. booting, heading and flowering, respectively.

Ten plants/cultivar were infected with each of BSMV + target, and BSMV:00, respectively, with three biological replicates. Inoculation on flag leaves was performed as described by Scofield et al. [11], while inoculation onto spikes was conducted by 5 times sliding with three gently pinched fingers from base to tip of the spikes.

### Total RNA extraction and cDNA synthesis

Total RNA was isolated from wheat spikelets or grains by using RNAiso-mate (TaKaRa, Dalian, China) and RNAiso Plus (TaKaRa, Dalian, China) followed by cold phenol/chloroform extraction three times. The quality and concentration of total RNA were determined with a Nanodrop ND-1000 spectrophotometer (Nano Drop Technologies, Wilmington, DE, USA). All the RNA samples were treated with RNase-free DNase I (TaKaRa, Dalian, China) prior to synthesizing cDNA as recommended by the manufacturer. For RT-PCR analysis of *PDS* and *1Bx14* transcript abundances, first-strand cDNA was synthesized using 500 ng of total RNA, oligo (dT) primer and MMLV reverse transcriptase (TaKaRa, Dalian, China).

For analysis of BSMV-VIGS in wheat spikes or grains, the spikelets or grains in the middle of BSMV infected spikes were collected at 2, 4, 8, 15, 20, 22 and 30dpi.

### Measurements of transcript abundances by qRT-PCR

Expression of the genes targeted for silencing was quantified by comparative quantitative real-time PCR (qRT-PCR). QRT-PCR was performed in triplicate for each RNA sample/primer combination. The amount of RNA in each reaction was normalized using primers specific for *actin*. The primer sequences used to detect each gene were as follows: *actin* forward, 5'-AAATCTGGCATCACACTTTCTAC-3'; *actin* reverse, 5'-GTCTCAAACATAATCTGGGTCATC-3';*PDS* forward P3, 5'-TGTCTTTAGCGTGCAAG-3', *PDS* reverse P4, 5'-GATGATTTCGGTGTCACT-3'; *1Bx14* forward P5, 5'-TAAGCGCCTGGTCCTCTTTGCG-3', *1Bx14* reverse P7, 5'-CGAAGGCGTAGTCTCGCTGGGG-3'. The positions of the primer set used for qRT-PCR are shown in Figure 1B. QRT-PCR was operated three times on iCycler iQTM Multi-Color Real Time PCR Detection System (Biorad, Hercules, CA, USA) using SYBR Green Master Mix (Biorad, http://www.bio-rad.com). The cycling conditions were as follows: 2 min at 95°C, followed by 45 cycles of 30 s at 95°C, 30 s at 55°C and 30 s at 72°C. In all cases, the relative expression of the targeted gene is presented as the expression level of this gene in silenced plants relative to that of the same gene in plants infected with BSMV:00, and the values of gene expression were the averages of three independent biological replicates. For each PCR, the specificity of the amplifications was validated and the threshold cycle above background was calculated using Bio-Rad iCycler software. PCR efficiency was close to 100%. Relative quantification of the gene transcript abundances was carried out applying an improved ΔΔ analysis [51]. Error bars in all figures showing qRT-PCR data indicated the standard deviations calculated from the original CT (cycle threshold) values. The P-values were estimated using hypothesis test (student test).

### Extraction of HMW-GSs in wheat seeds and analysis by SDS-PAGE

To determine the efficacy of *1Bx14* silencing at protein level, grains in the middle of wheat spikes from BSMV:1Bx14, BSMV:HMW or BSMV:00 inoculated plants were collected at 20 dpi, respectively, and HMW-GSs were extracted from one grain of each sample with 200ul solution (50%(v/v) 1-propanol, 0.625 mol/L Tris–HCl (pH = 6.8), 10% SDS, 2% DDT, 20% Glycerin and 0.2% Bromophenyl blue). Equal volumes (20 μl) of each protein sample were loaded and separated by SDS-PAGE with 5% stacking and 12% resolving polyacrylamide. Each treatment was performed with three independent biological replicates.

### Measurement of total proteins, total glutenin polymeric proteins and glutenin macropolymeric proteins of wheat grains

Each grain sample of approximate 0.2 g was milled and equipped with a 0.5 mm screen to obtain wholemeal flour. Total glutenin polymers free from monomeric proteins and glutenin macropolymeric proteins (50% 1-propanol insoluble fraction) of each wholemeal flour sample were isolated as previously described by Xu et al. [44]. The protein contents of total glutenin polymer, glutenin macropolymer or wholemeal flour were determined by micro-Kjeldahl (Foss Tecator AB, Hoganas, Sweden) analysis. The amount of proteins was estimated as N × 5.7. Each treatment was performed with three independent biological replicates.

## Authors’ contributions

MM carried out the VIGS, genomic analysis and qRT-PCR, created the figures, and ran the SDS-PAGE. YY supported the VIGS analysis and raised the plant material. LH provided the BSMV vectors, technical advice, and edited the manuscript. MC participated in modification of the manuscript. HZ designed the experiments and coordinated their implementation. MM and HZ participated in drafting the manuscript. All authors read and approved the final manuscript.

## Authors' information

MM, YY, and HZ: State Key Laboratory of Crop Stress Biology for Arid Areas, College of Life Sciences, Northwest A & F University, Yangling, 712100, Shaanxi, China.

LH: Department of Plant Sciences & Plant Pathology, Montana State University, Bozeman, MT59717, USA.

MC: USDA-ARS and Department of Entomology, Kansas State University, Manhattan, KS66506, USA.

## Supplementary Material

Additional file 1 **Identity of the 176-bp fragment of*1Bx14*with the full-length of*HMW-GS*gene*1Ax1, 1Bx14, 1By15, 1Dx2*and*1Dy12*in wheat cultivars Xiaoyan 6 and Shaanyou 225.** Click here for file

Additional file 2 **Nucleotide sequence alignment of the*asX*or the*asY*with their corresponding elements in other x-type (upper region) or y-type (lower region)*HMW-GS*genes expressed in the wheat variety, respectively;*asX*and*asY*represent sequences highly homologous to most of all x-type or y-type*HMW-GS*genes, respectively.** Nucleotides conserved in all sequences are represented by ‘*’. The alignment was conducted using the Clustal W program. Click here for file
