# Supplementary material for: Virus-induced gene-silencing in wheat spikes and grains and its application in functional analysis of HMW-GS-encoding genes
Source: BMC Plant Biol. 2012 Aug 10;12:141. doi: 10.1186/1471-2229-12-141 (PMC3462119; doi:10.1186/1471-2229-12-141)
Supplement: Additional file 1 — Identity of the 176-bp fragment of1Bx14with the full-length ofHMW-GSgene1Ax1, 1Bx14, 1By15, 1Dx2and1Dy12in wheat cultivars Xiaoyan 6 and Shaanyou 225. [file 1471-2229-12-141-S1.pdf]

Additional file 1: Identity of the 176-bp fragment of *1Bx14* with the full-length of *HMW-GS* gene *1Ax1, 1Bx14, 1By15, 1Dx2* and *1Dy12* in wheat cultivars Xiaoyan 6 and Shaanyou 225.

HMW-GS gene	Nucleotide identity (%)	Longest match between the fragment and *1Ax1*, *1Bx14*, *1By15*,*1Dx2* or *1Dy12*（bp）
1Ax1	60.45	19
1Bx14	100	176
1By15	69.32	16
1Dx2	82.95	16
1Dy12	70.06	18
