# Supplementary material for: Virus-induced gene-silencing in wheat spikes and grains and its application in functional analysis of HMW-GS-encoding genes
Source: BMC Plant Biol. 2012 Aug 10;12:141. doi: 10.1186/1471-2229-12-141 (PMC3462119; doi:10.1186/1471-2229-12-141)
Supplement: Additional file 2 — Nucleotide sequence alignment of theasXor theasYwith their corresponding elements in other x-type (upper region) or y-type (lower region)HMW-GSgenes expressed in the wheat variety, respectively;asXandasYrepresent sequences highly homologous to most of all x-type or y-typeHMW-GSgenes, respectively. Nucleotides conserved in all sequences are represented by ‘*’. The alignment was conducted using the Clustal W program. [file 1471-2229-12-141-S2.pdf]

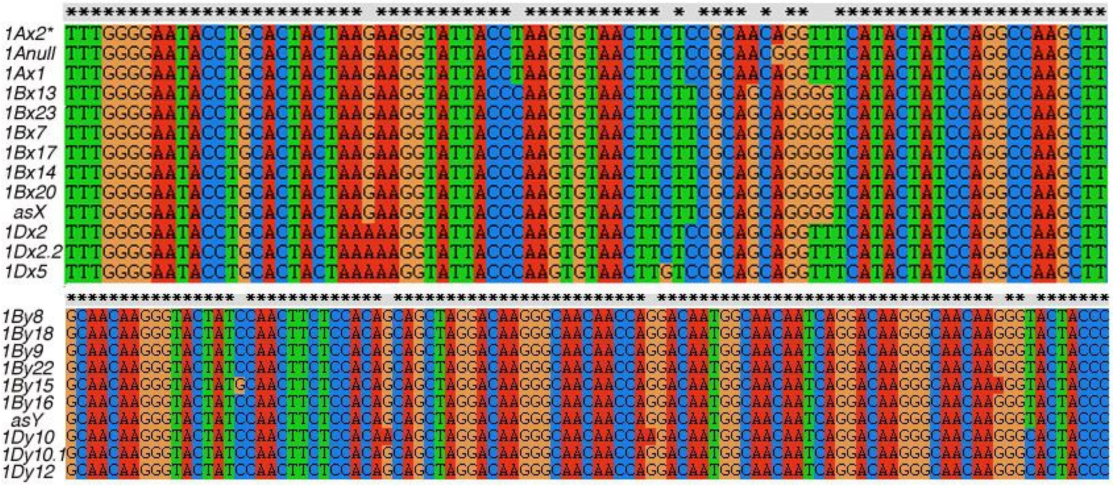

Additional file 2:Nucleotide sequence alignment of the *asX* or the *asY* with their corresponding elements in other x-type (upper region) or y-type (lower region) *HMW-GS* genes expressed in the wheat variety, respectively; *asX* and *asY* represent sequences highly homologous to most of all x-type or y-type *HMW-GS* genes, respectively. Nucleotides conserved in all sequences are represented by ‘*’. The alignment was conducted using the Clustal W program.
